# Assessing biologic/toxicologic effects of extractables from plastic contact materials for advanced therapy manufacturing using cell painting assay and cytotoxicity screening

**DOI:** 10.1038/s41598-024-55952-3

**Published:** 2024-03-11

**Authors:** Ina Pahl, Axel Pahl, Armin Hauk, Dana Budde, Sonja Sievers, Lothar Fruth, Roberto Menzel

**Affiliations:** 1 Sartorius Stedim Biotech GmbH, August-Spindler-Str. 11, 37079 Göttingen, Germany; 2 Compound Management and Screening Center, MPI of Molecular Physiology, Otto-Hahn-Str. 11, 44227 Dortmund, Germany; 3 Tox Expert GmbH, An der Feldscheide 1, 37083 Göttingen, Germany

**Keywords:** Cell painting assay (CPA), Cell and gene therapy, Advanced therapy medicinal products (ATMP), Extractables and leachables, Single-use systems, Process equipment-related leachables (PERL), Biotechnology, Cell biology

## Abstract

Plastic components are essential in the pharmaceutical industry, encompassing container closure systems, laboratory handling equipment, and single-use systems. As part of their material qualification process, studies on interactions between plastic contact materials and process solutions or drug products are conducted. The assessment of single-use systems includes their potential impact on patient safety, product quality, and process performance. This is particularly crucial in cell and gene therapy applications since interactions with the plastic contact material may result in an adverse effect on the isolated therapeutic human cells. We utilized the cell painting assay (CPA), a non-targeted method, for profiling the morphological characteristics of U2OS human osteosarcoma cells in contact with chemicals related to plastic contact materials. Specifically, we conducted a comprehensive analysis of 45 common plastic extractables, and two extracts from single-use systems. Results of the CPA are compared with a standard cytotoxicity assay, an osteogenesis differentiation assay, and in silico toxicity predictions. The findings of this feasibility study demonstrate that the device extracts and most of the tested compounds do not evoke any measurable biological changes on the cells (induction  ≤ 5%) among the 579 cell features measured at concentrations  ≤ 50 µM. CPA can serve as an important assay to reveal unique information not accessible through quantitative structure–activity relationship analysis and vice versa. The results highlight the need for a combination of in vitro and in silico methods in a comprehensive assessment of single-use equipment utilized in advanced therapy medicinal products manufacturing.

## Introduction

### Study objectives

In this study we are presenting a cell painting assay (CPA) and cytotoxicity screening to assess biological effects of chemicals related to plastic contact materials on human cells. The assay was performed with a human cell line (U2OS). The selection of compounds included typical extractables that can be expected for plastics used in single-use systems (SUS) and compounds with known toxicological risks. Common cellular process parameters and cytotoxic effects of 45 extractables were investigated on the U2OS human cells. Additionally, we tested two complex solutions: extracts from a polyethersulfone (PES) membrane filter and a silicone tube. The results are compared to a standard cell culture growth test (ASTM E3231-19). Finally, we screened the organic compounds for toxicological endpoints using established in silico quantitative structure-activity relationship (QSAR) tools.

### Single-use technology in biopharmaceuticals and advanced therapy processes

The biopharmaceutical industry relies on single-use technology (SUT) for manufacturing drug products (DP), such as biologics and small molecule drugs. This technology offers several advantages, such as flexibility in design and manufacturing space and low risk for contamination^1,2^. A major concern for SUT are process equipment-related leachables (PERLs) migrating from the plastic construction materials into the process stream, potentially reaching the final DP as leachables. Legally, contact materials in pharmaceutical applications shall be sufficiently “inert”^3^. Authorities require the evaluation of PERLs to assess their impact on the process performance, patient health, and DP quality to demonstrate the suitability of the single-use device^4–6^.

Similar challenges arise for advanced therapy (AT) applications where, for example, cells are taken from a donor or patient, modified ex vivo using SUS, and subsequently administered to the patient^7^. The qualification of single-use devices, used as ancillary materials and not intended to become part of the final AT product, is comparable to qualifications in biopharmaceutical processes^7–9^. USP 〈1043〉 (United States Pharmacopoeia) provides a tiered approach for risk assessment of ancillary material selection, which prefers highly qualified materials for manufacturing AT products^8^. Currently, there is a general lack of data on compatibility of materials with cells and products from advanced therapy medicinal products (ATMP) manufacturing^10^.

### Patient exposure

Unique challenges arise when considering the safety of PERLs in AT because various types of exposure must be considered. Patient exposure risks include the PERL exposure to the cells during the ex vivo manipulation and processing, and the direct infusion of the liquid and the cellular phase^7^. A practice to determine PERL exposure, i.e., measurement of trace contaminants adsorbed on cells and/or dissolved in the liquid phase, has not yet been established. An elegant alternative is to model the exposure of PERLs onto the therapeutic cells and the patient using available extractables data of SUS. This approach considers the physical parameters associated with PERL uptake by cells and the specific processing conditions^4,11–13^. There are established concepts for realistic exposure calculations that take into account the sources and sinks of PERLs^14–17^. These might also be applicable to ATMP manufacturing. However, in AT processes, impurity removal is less efficient and is typically achieved through simple washing steps^7^.

Methods are not yet established to investigate the biological effects of PERLs on therapeutic cells, such as impaired cell proliferation or viability. Budde et al.^18^ reported detrimental effects on T-cell growth and loss of potency caused by compounds migrating from plastic materials used in AT applications. Another highly relevant problem expressed by various stakeholders, including authorities, is the formation of degenerated cells due to ex vivo manipulations of cells in the artificial environment of plastics, as associated with SUS. Degenerated cells can lead to critical health issues for the patient, such as tumor formation^19^.

### Biocompatibility testing

Several biological tests are used to complement the evaluation for suitability of SUS besides extractables safety assessments. Mandatory tests include USP 〈87〉 and ISO 10993-5 cytotoxicity tests (International Organization for Standards)^20,21^. To address the suitability-for-use of SUS in biopharmaceutical upstream processes, specific cell tests were developed by DECHEMA (Gesellschaft für Chemische Technik und Biotechnologie e.V.) and ASTM (American Society for Testing and Materials). These tests are designed to evaluate the compatibility of SUS with host cells, e.g., Chinese hamster ovary (CHO) cells, for the production of monoclonal antibodies (ASTM E3231-19)^22,23^.

In vivo animal studies can detect a broad spectrum of systemic effects on the tested organism. In contrast, in vitro test systems are very specific but can typically detect only one aspect of a toxicological endpoint. Notably, the relevance of in vivo animal test endpoints to assess ex vivo manipulated human cells in ATMP is questionable; effects measured under in vitro conditions are likely more relevant^24,25^. SI-Table 1 shows common test methods and quality attributes for the qualification of SU materials used as equipment in biopharmaceutical and AT manufacturing. Consideration should also be given to the limited transferability of results to humans and ethical aspects of animal testing (e.g., USP 〈88〉) for safety evaluations in both applications^24–28^.

### Toxicological risk assessment

Assessment methods are established for therapeutic biological drugs and pharmaceuticals^6,29–32^. A toxicological risk assessment of a compound uses health-based exposure limits, such as the permitted daily exposure (PDE) value in the regulation of pharmaceutical products^33,34^. Similar methods are published for medical devices in the guidance document ISO 10993-17, which uses the term “tolerable intake^35^”. Toxicologists face challenges when extrapolating results from oral studies with animals to parenteral pathways. This is due to the need for careful consideration of appropriate factors for route-to-route extrapolation, taking into account the compounds' bioavailability^29,33^. It is essential to handle this process with care to avoid overlooking significant route-specific effects^36–38^.

Nowadays, in vitro studies are widely accepted in regulatory safety testing for the toxicological endpoints of cytotoxicity, skin irritation, eye irritation, skin sensitization, mutagenicity, and the identification of endocrine disruptors^39^. In consequence, multiple in vitro studies with cells are often required to cover one toxicological endpoint—a popular example is skin sensitization^40^. We employ QSAR methods to screen the toxicological properties of tested extractables. This involves analyzing compounds with similar structures through read across information to evaluate the toxicological properties.

Test systems designed to address direct effects of chemicals—i.e., extractables or leachables from SUS—might overcome limitations in deriving PDE values, making them an interesting option for toxicological assessments.

### Cell painting assay

The Cell Painting assay (CPA) is a high-content and image-based method that measures phenotypic changes in mammalian cells, including human cells, by employing multiplexed fluorescent dyes to stain various cellular components or organelles, allowing the identification of small-molecule bioactivity in perturbed or restored cellular phenotypes^41^. Studying cell morphology offers a visual insight into molecular and physiological processes, reflecting the cell's function, health, differentiation, and response to chemicals, including drugs or contaminations. CPA enables the observation of cellular morphology/phenotype changes and is, therefore, applicable for uncovering biological information about the cellular state and potential perturbations without the need for a prior target hypothesis^42^. It is not limited to a phenotype of interest in a given assay and covers a wide bioactivity space^42,43^. By comparing morphological profiling data from reference compounds with known biological activities, it is feasible to identify biological pathways and modes of action for newly tested compound^43–46^.

CPA is used in pharmaceutical research to support target identification for drugs^47,48^, and to evaluate human-relevant toxicity mechanisms with adherent human cells^49,50^. Primary immune cells, such as peripheral blood mononuclear cells (PBMCs), were used to reveal molecular health-associated phenotypes with non-adherent cells^41^. CPA is considered as a useful human-relevant new approach methodology (NAM) and a valuable technology to reduce animal testing. However, it should be supplemented with other NAM and not applied as standalone methodology^51–53^.

In our study we used one type of human cells to investigate exemplarily potential effects on human cells used in ATMP processes. The evaluation of several hundreds of cell features is a significant advantage because we assume that most of these features are related to common biological functions and parameters which are relevant for any human cell type. This include morphological integrity, energy production, protein synthesis, reproduction, and cell growth.

However, it is important to note certain limitations of the CPA:

- Similar to other cell-based assays, compound stability in the assay system and bioavailability in the cells is required.
- Activity in the CPA relies on changes in the cell’s morphology. Only compounds acting on targets/pathways whose modulation induces a morphological change will give an observable effect (induction) in the CPA.
- Comparing intensities of profiles with low similarity does not allow conclusions about different compound activities since the modulation of different targets may induce different extents of morphological effects.
- Although, the implementation of the CPA employed in this study analyzes 579 stable morphological features (see experimental setup of CPA) associated to desired and undesired human cell effects, using just one cell type in this in vitro CPA cannot fully assess effects on all cell types or the entire human body. Testing other cell types and specific parameters that are tailored to each cell type is recommended but would exceed the scope of this feasibility study.

## Material and methods

### Tested compounds and extracts

Solvents and tested chemicals were of synthesis grade or better. Stock solutions (10 mM) of the test compounds were prepared in dimethyl sulfoxide (DMSO, CAS 67-68-5, Serva, Cat No, 20385). The selected molar concentration range for the CPA is 10 to 50 µM.

In total, 45 compounds, including several known extractables, were selected for screening by the CPA. They are provided with information on their application or formation in SI-Table 2. Furthermore, two 50% ethanol extracts from an extraction study of two SU components—a sterilizing-grade filter and silicone tubing—were subjected to the test. The 50% ethanol of the SU filter and tubing contained a mixture of  > 20 different extractable compounds with concentrations between 0.1 and up to 2 µg/ml for the filter and up to 11 µg/ml for the tube extract. Leachable concentrations are typically much lower than extractables, making the selected compound concentrations relevant for cell impact investigation.

Comprehensive details on compound selection and extractions are provided also in SI-Table 2. Practical limitations of compound selection arise from the requirement that the compounds must be accessible to the cells, i.e., can be extracted and dissolved in the media. Therefore, almost all compounds tested have a log *P* ≤ 5. log *P* is the logarithm of the partition coefficient between water and octanol (K_o/w_).

### Experimental setup of CPA

The assay follows closely the method described by Bray et al.^43^ Initially, 5 µL U2OS medium were added to each well of a 384-well plate (PerkinElmer CellCarrier-384 Ultra). Subsequently, U2OS cell were seeded with a density of 1600 cells per well in 20 µL medium. The plate was incubated for 10 min at room temperature (RT), followed by an additional 4 h incubation (37 °C, 5% CO_2_). Compound treatment was performed with the Echo 520 acoustic dispenser (Labcyte) at final concentrations of 50, 30, or 10 µM and, if necessary, at lower concentrations. Incubation with compound was performed for 20 h (37 °C, 5% CO_2_). Subsequently, mitochondria were stained with Mito Tracker Deep Red (Thermo Fisher Scientific, Cat. No. M22426). The Mito Tracker Deep Red stock solution (1 mM) was diluted to a final concentration of 100 nM in prewarmed medium. The medium was removed from the plate leaving 10 µL residual volume and 25 µL of the Mito Tracker solution were added to each well. The plate was incubated for 30 min in darkness (37 °C, 5% CO_2_). To fix the cells, 7 µl of 18.5% formaldehyde in PBS were added, resulting in a final formaldehyde concentration of 3.7%. Subsequently, the plate was incubated for another 20 min in darkness (RT) and washed three times with 70 µL of PBS (Biotek Washer Elx405). Cells were permeabilized by addition of 25 µL 0.1% Triton X-100 to each well, followed by 15 min incubation (RT) in darkness. The cells were washed three times with PBS leaving a final volume of 10 µL. To each well 25 µL of a staining solution were added, which contains 1% BSA, 5 µL/mL Phalloidin (Alexa594 conjugate, Thermo Fisher Scientific, A12381), 25 µg/ml Concanavalin A (Alexa488 conjugate, Thermo Fisher Scientific, Cat. No. C11252), 5 µg/ml Hoechst 33342 (Sigma, Cat. No. B2261-25 mg), 1.5 µg/ml WGA-Alexa594 conjugate (Thermo Fisher Scientific, Cat. No. W11262) and 1.5 µM SYTO 14 solution (Thermo Fisher Scientific, Cat. No. S7576). The plate is incubated for 30 min (RT) in darkness and washed three times with 70 µL PBS. After the final washing step, the PBS was not aspirated. The plates were sealed and centrifuged for 1 min at 50 × g.

**Figure 1:**
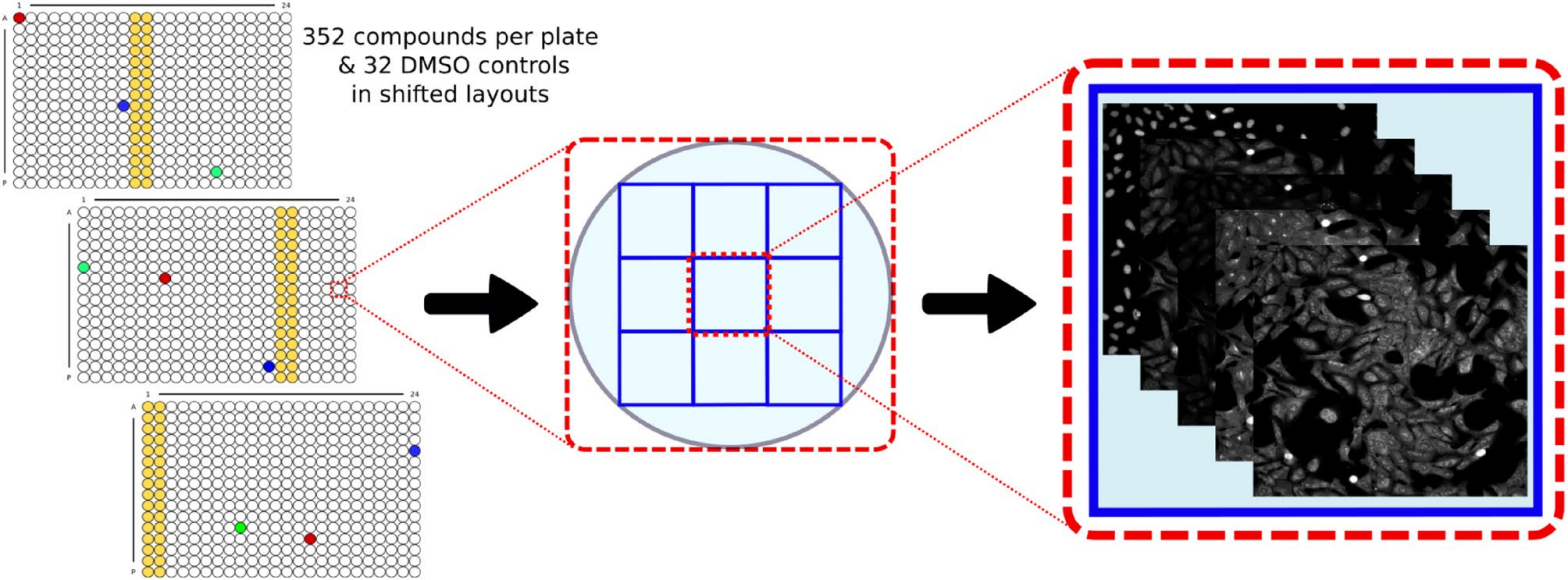
Plate test layout and imaging. High-Content Screening system parameters: 5 channels (DAPI: Ex350-400/Em410-480; FITC: Ex470-500/Em510-540; Spectrum Gold: Ex520-545/Em560-585; TxRed: Ex535-585/Em600-650; Cy5: Ex605-650/Em670-715) with 9 sites per well and 20 × magnification (binning 2).

The plates were prepared in triplicates with shifted layouts to reduce plate effects and imaged using a Micro XL High-Content Screening System (Molecular Devices) as shown in Figure 1.

The images generated were processed with the CellProfiler package (https://cellprofiler.org/, version 3.0.0) on a computing cluster of the Max Planck Society to extract 1716 cell features per microscope site. The data was further aggregated as medians per well (9 sites → 1 well), then over the three replicates.

Further analysis was performed with custom Python (https://www.python.org/) scripts using the Pandas (https://pandas.pydata.org/) and Dask (https://dask.org/) data processing libraries as well as the Scientific Python (https://scipy.org/) package (separate publication to follow).

Out of the initial 1716 features, a subset of 579 highly reproducible and robust features was determined using a procedure based on the criteria described by Woehrmann et al.^54^ Two biological repeats of one plate containing reference compounds were analysed. For every feature, its full profile over each whole plate was calculated. If the profiles from the two repeats showed a similarity ≥ 0.8 (see below), the feature was added to the set. This procedure was performed once and served as the basis for all subsequent analyses.

The phenotypic profiles were compiled from the Z-scores of all individual cellular features, where the Z-score is a measure of how far away a data point is from a median value.

Specifically, Z-scores of test compounds were calculated relative to the Median of DMSO controls. Thus, the Z-score of a test compound defines how many MADs (Median Absolute Deviations) the measured value is away from the Median of the controls as illustrated by the following formula:

$$Z-score=\frac{{value}_{meas.}-{Median}_{Controls}}{{MAD}_{Controls}}$$

**Figure 2:**
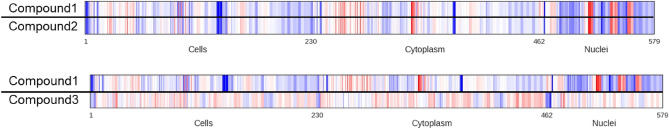
Above: An example for two compounds with highly similar profiles (96% biosimilarity); below: An example for two compounds with low similarity profiles (0% Biosimilarity): In each case, colored bands represent the Z-scores of individual features.

The phenotypic compound profile is then determined as the list of Z-scores of all features for one compound and is visualized via heatmap (examplarily see Fig. 2).

In addition to the phenotypic profile, an induction value was determined for each compound as the fraction of significantly changed features, in percent:

$$Induction\left[ \% \right] = \frac{{number\;of\;features\;with\;abs.\;values > 3}}{{total\;number\;of\;features}}$$

Similarities of phenotypic profiles (termed Biosimilarity) were calculated from the correlation distances (CD) between two profiles (https://docs.scipy.org/doc/scipy/reference/generated/scipy.spatial.distance.correlation.html):

$$CD=1-\frac{\left(u-\overline{u }\right) \cdot (v-\overline{v })}{{\Vert (u-\overline{u })\Vert }_{2}{\Vert (v-\overline{v })\Vert }_{2}}$$

where $\overline{x }$ is the mean of the elements of $x$, $x\cdot y$ is the dot product of $x$ and $y$, and ${\Vert x\Vert }_{2}$ is the Euclidean norm of $x$:

$${\Vert x\Vert }_{2}=\sqrt{{x}_{1}^{2}+{x}_{2}^{2}+\dots +{x}_{n}^{2}}$$

The biosimilarity is then defined as:

$$Biosimilarity=1-CD$$

Biosimilarity is expressed as a percentage (0–100), and values smaller than 0 are set to 0.

In addition to calculating biosimilarity between the full morphological profiles of two Cell painting measurements, Pahl et al.^44^ developed an approach to assign similarity to biological clusters by comparing sub-profiles.

**Figure 3:**
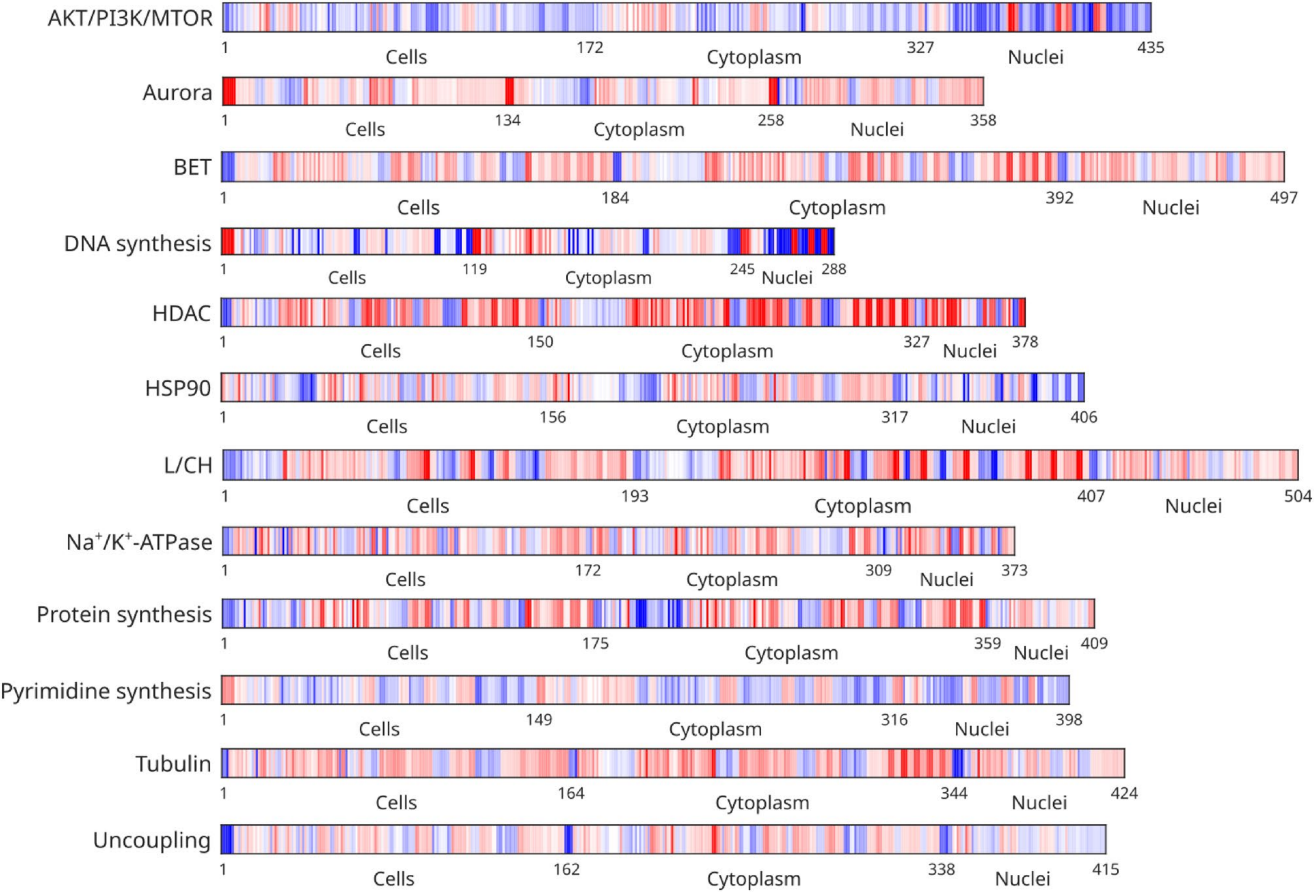
List of currently identified clusters and their median profiles. By comparing the median cluster profiles to the matching sub-profiles of measured compounds (only profiles of the same length can be compared for similarity), a biosimilarity to each cluster can be calculated.

In essence, a set of 12 biological clusters was defined from Cell painting measurements with confirmed activity on these clusters. By considering only the features with similar values from the group of measurements for each cluster, a representative median profile was calculated for each cluster. These representative median profiles are of different length and shape for each cluster (Fig. 3).

This experimental setup represents a proven practice when performing CPAs to detect morphological changes of adherent human U2OS cells using a serum-containing defined cell culture medium^55,56^. Possible quenching effects with the used dyes in the CPA are considered of low concern because the fluorescent dyes are not in contact with the tested compounds during incubation.

### Osteogenesis inhibition assay (OIA)

The osteogenesis inhibition assay was performed as described elsewhere^57^. Briefly, murine mesodermal stem cells (C3H10T1/2) were stimulated with purmorphamine (a Hedgehog agonist) to induce the expression of the osteogenesis marker alkaline phosphatase. After treatment with compounds for 96 h, the expression of alkaline phosphatase was quantified using a luminogenic substrate (CDP-Star, Roche), which reveals whether a compound is a developmental pathway inhibitor of, e.g., the Hedgehog (Hh) signaling pathway or other related pathways.

### Cytotoxicity testing with CHO cells

An IgG1-producing Chinese hamster ovary (CHO) cell line (CHO DG44 from Sartorius Stedim Cellca GmbH, Germany) was cultivated in chemically defined media (ActiCHO SM, Cytiva, USA). The cell line was adapted to grow in suspension without animal-derived supplements. Spiking experiments were carried out after cultivation as described elsewhere^58^. This method determines effective concentrations (EC_50_) that cause 50% inhibition of cell growth. Data evaluation was carried out according to the method described in ASTM E3231-19, “Standard Guide for Cell Culture Growth Assessment of Single-Use Material^23^”. This test defines best practices using serum-free, chemically-defined cell culture medium to prevent false negative results.

### QSAR analysis

The software “Vega 1.2.0” (www.vegahub.eu/) was used for the prediction of mutagenicity (Ames test) by QSAR analysis. The software “QSAR Toolbox 4.5” (https://qsartoolbox.org) gathered experimental toxicological data such as in vitro genotoxicity studies and made predictions of toxicity using 15 models with different prediction profiles for protein binding and deoxyribonucleic acid (DNA) alerts, mutagenicity, and irritation (https://qsartoolbox.org). The selected models are suitable to reveal structure motifs suspected to form covalent modifications of proteins or DNA, posing a significant risk in AT and other biopharmaceutical processes. Li et al*.*^6^ give examples of such structures. The toxic hazard classification by Cramer may also indicate such risks^59^.

## Results and discussion

### Cell painting assay

We tested compounds which are primarily extractables of SUS one can expect in aqueous solutions, e.g. used in biopharmaceutical or in ATMP processes. Most of the examined compounds did not show any significant effect on the 579 morphological features evaluated at concentrations exceeding those encountered in extractables studies of SU devices. We consider compounds with an induction  ≥ 5% active in the CPA^44^. Remarkably, only a limited subset comprising 4 out of 45 extractable substances demonstrated an induction level surpassing the predefined 5% threshold in the context of the assessed human cells. The results are summarized in Table 1. The four compounds are an antistatic agent *N*-lauryldiethanolamine, an antioxidant degradant bis(2,4-di-*tert*-butylphenyl)phosphate (bD*t*BPP) known for its cell growth inhibition, a starting material for non-ionic surfactants or antioxidants 4-n-nonylphenol, and a photoinitiator diphenyl(2,4,6-trimethylbenzoyl)phosphine oxide (TPO). The two organic extraction solutions of the sterilizing-grade filter and the silicon tubing consisting of approximately (approx.) 20 extractables showed no effect in the CPA﻿.

**Table 1:** CPA and osteogenesis inhibition assay results.

Active compound	Induction in CPA [%] (concentration)	Highest similarity to biological clusters (≥ 65%) [%]	Cell count [% of control] (concentration)	Osteogenesis inhibition assay [IC50]
*N-*Lauryldiethanolamine	61 (3 µM)	Cholesterol homeostasis (88%); lysosomotropic	11 (50 µM)	≥ 10 µM (toxic)
bD*t*BPP	17 (50 µM)	DNA synthesis (65%)	87 (50 µM)	6.8 µM
4-n-Nonylphenol	17 (30 µM)	DNA synthesis (70%)	91 (50 µM)	3.8 µM
TPO	11 (30 µM)	HDAC (77%); Weak lysosomotropic	94 (50 µM)	5 µM

CPA for the four extractables showing an induction > 5%. *Induction in CPA* describes the bioactivity of the compound and is defined as the percentage of altered features compared to the controls. *Highest Similarity to Biological Clusters* is the assignment of the compound to *reference compounds with a confirmed biological activity based on similarities in cluster features*^44^*.* *Cell Count* is the ratio of the number of detected cells in the test sample and the number of cells in the control sample in which the cells divide once within the incubation time of 20 h. A cell count of 50% means a cell arrest was observed: no cell division took place in the test sample. The relative cell count can be considered as the sum of different effects that may end up and result in growth inhibition. *Osteogenesis Inhibition Assay* is the half maximal inhibitory concentration (IC_50_) describes an inhibitory effect to a specific function, e.g., alkaline phosphatase expression and is tested with the osteogenesis inhibition assay. It was performed for all tested compounds.

*N*-Lauryldiethanolamine was the only compound with a strong inhibitory effect on cell growth, starting from 30 µM. An induction of 61% was found at the lowest test concentration of 3 µM (additional lower concentration tested) with a predicted effect on cholesterol homeostasis. Cholesterol homeostasis describes processes that maintain a steady state level of cholesterol in the cells. Furthermore, the compound shows some lysosomotropic behavior at 30 µM. Lysosomotropic active compounds do not have a designated target in the cell but rather migrate into the cellular lysosome due to a weak alkaline nature^46,60^. Upon protonation within the lysosome, the molecules cannot escape and accumulate, resulting in a lysosomal burst triggering apoptotic signaling. Drugs with a log *P* > 2 and *p*Ka between 6.5 and 11 commonly cause lysosomal accumulation (*N-*Lauryldiethanolamine: calc. log *P*: 3.2, calc. *p*Ka 9.2)^61^. Morphological profiling has captured small morphological changes upon a given perturbation before it reaches toxic levels. *N*-Lauryldiethanolamine was toxic at a concentration of  ≥ 10 µM in the osteogenesis inhibition assay and excluded from further dose-response measurement.

The compounds bD*t*BPP, 4-n-nonylphenol, and TPO show inductions below 20%. Fortunately, these three active compounds are commonly not linked to SU construction materials nowadays, or their level is reduced and controlled (e.g., bD*t*BPP)^62^. Compounds bD*t*BPP and 4-n-nonylphenol show low similarity to the DNA synthesis cluster, and TPO shows medium similarity to the histone deacetylase (HDAC) cluster. TPO also showed a lysosomotropic behavior in line with a log *P* > 2 (TPO: calc. log *P*: 4.8). Surprisingly, bD*t*BPP revealed no impact on cell growth for the U2OS cells (see next chapter). Subsequent analysis in the osteogenesis inhibition assay revealed that three of the four compounds inhibit the osteogenesis pathway.

**Figure 4:**
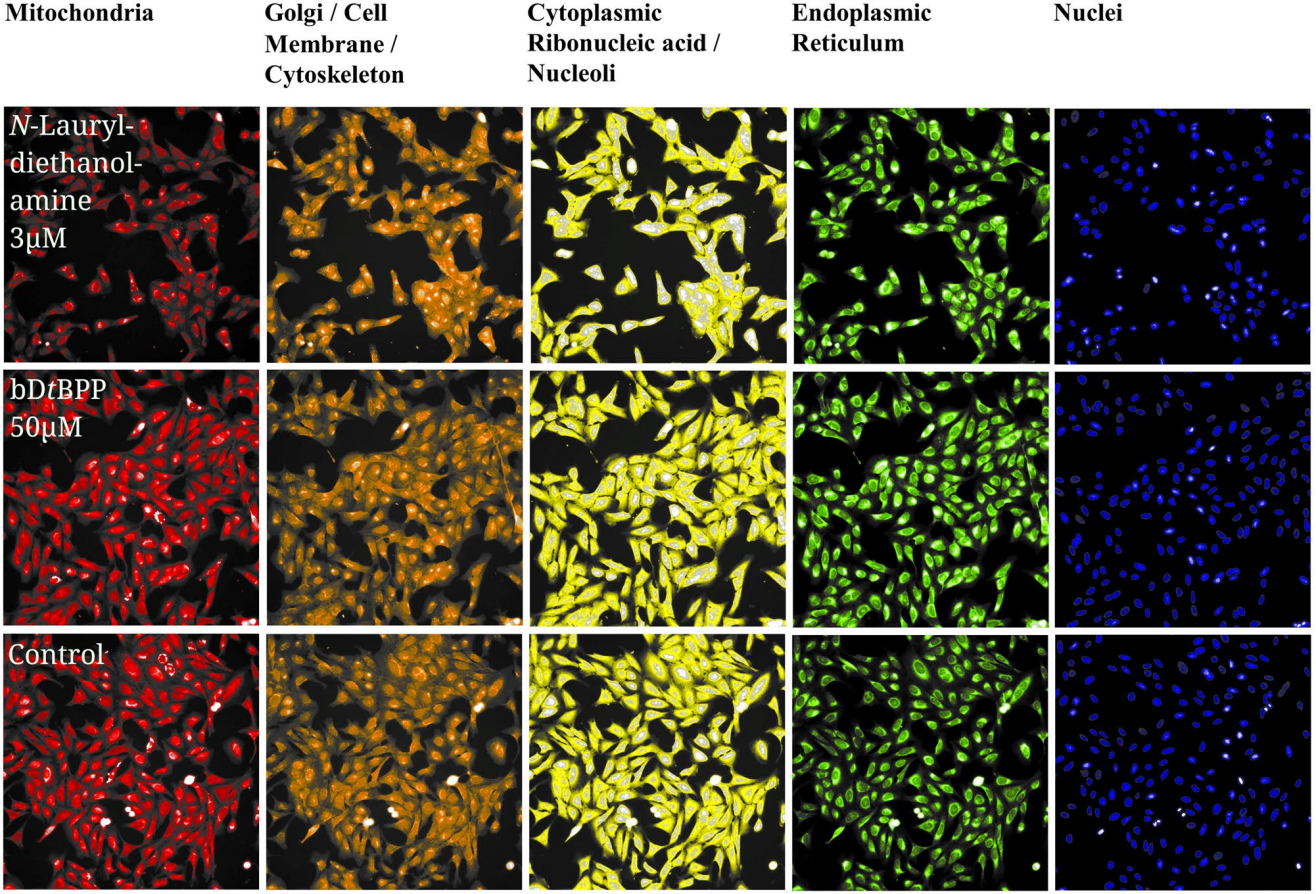
The CPA in U2OS cells. The top row shows cells after treatment with *N*-Lauryldiethanolamine; the middle row shows cells after treatment with bD*t*BPP; and the bottom row shows cells after treatment with the control (DMSO). The columns display the five channels imaged in the CPA-focusing cellular compartments: Mitochondria (MitoTracker Deep Red); Golgi/Cell Membrane/Cytoskeleton (Wheat-germ agglutinin-Alexa Fluor 555/Phalloidin-Alexa Fluor 568); Cytoplasmic RNA/Nucleoli (SYTO 14 green fluorescent nucleic acid stain); Endoplasmic Reticulum (Concanavalin A-Alexa Fluor 488); Nuclei (Hoechst 33342). Images were captured at 20 × magnification.*RNA: ribonucleic acid.

**Figure 5:**
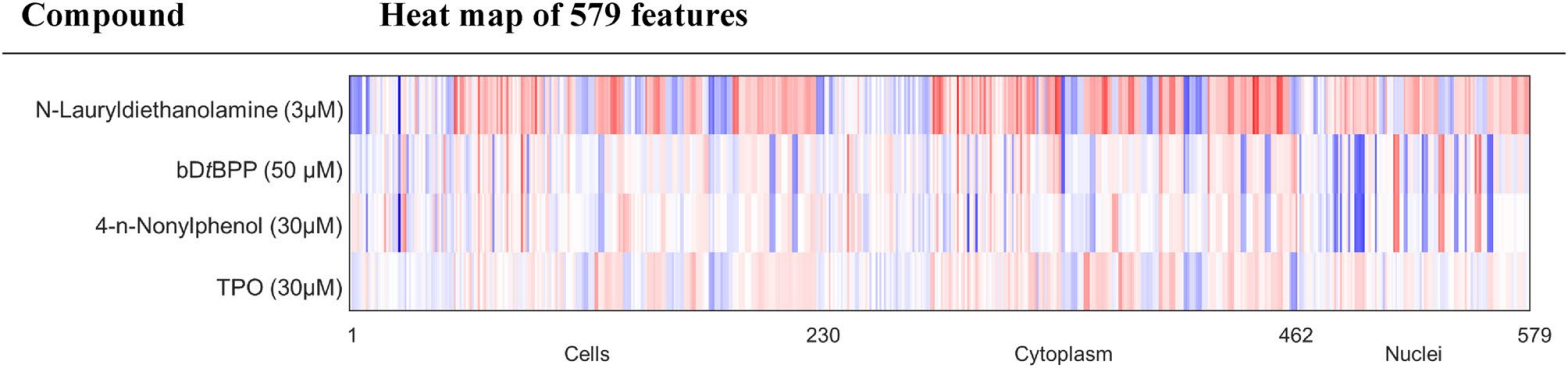
Heatmap of compounds (with molarity) active in the CPA showing scored cell profiles from 579 features. Each colored band represents one Z-score of a feature. Blue color: decreased feature, red color: increased feature.

Figure 4 shows microscopic images of the cell organelles after treatment with *N*-Lauryldiethanolamine or bD*t*BPP compared to the control sample (DMSO). When examining the images, *N*-Lauryldiethanolamine differs significantly from the control, which cannot be easily recognized for bD*t*BPP. Based on this visual analysis, it is apparent that these two substances have different profiles. The automated image analysis carried out for CPA reveals the distinct profiles (Fig. 5), with the one for *N*-Lauryldiethanolamine being more intense than those for the other three compounds In Fig. 4 the measured features are assigned to the compartments defined by CellProfiler using the five imaged channels.

**Figure 6:**
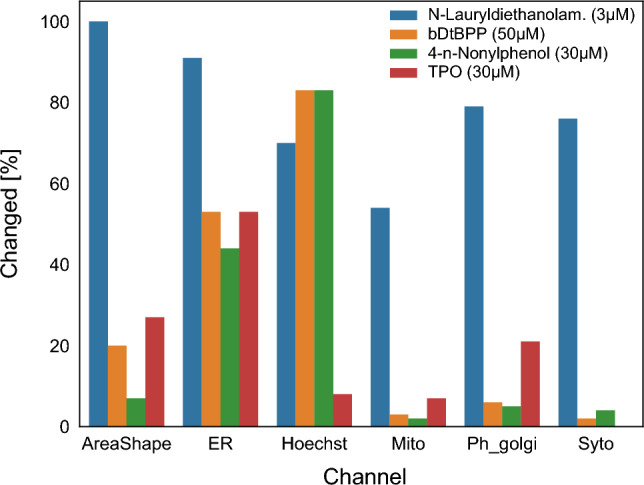
Percentage of changed imaged channels for CPA-active compounds. ER: Endoplasmic Reticulum; Hoechst: Nuclei; Mito: Mitochondria; Ph_golgi: Golgi/Cell Membrane/Cytoskeleton; Syto: Cytoplasmic RNA/Nucleoli.

During CPA evaluation the cellular compartments are imaged in five different channels. In Fig. 6, the percentage of changed features that depend on individual channels is shown. In addition, the figure contains the changed features for the AreaShape group. The channels related to the endoplasmic reticulum (ER) and nuclei (Hoechst) experience the highest degree of change for the four CPA active compounds. The percentage of changed features is above 40% with the exception of TPO with approx. 5% for nuclei. *N*-Lauryldiethanolamine induced at the concentration of 3 µm a percentage of changed features of approx. 50 to 90% for all channels. The channels for mitochondria (Mito), golgi/cell membrane/cytoskeleton—compartments (Ph_golgi), and cytoplasmic RNA/nucleoli are changed by the other three compounds below 20%. The AreaShape group is related to the shape of the cells. *N*-Lauryldiethanolamine changed all features related to this group, and bD*t*BPP or TPO changed approx. 20 or 30% of the features, respectively.

### Comparison of CPA results with CHO toxicity testing data

Toxicity testing of selected extractables has been carried out with a commonly used CHO cell line according to ASTM E3231-19 to assess the potential impact of these compounds on biopharmaceutical cell culture performance. Table 2 summarizes the results of spiking experiments using an antibody-secreting CHO DG44 cell line with extractables that showed activity in the CPA. In contrast to the CPA, experiments were carried out without serum supplementation and in chemically defined cultivation medium to sensitively detect the endpoint cell growth. Compared to the U2OS cell line used in the CPA, the CHO cell line with the given test setup (e.g., serum-free medium, incubation time of 72 h) showed to be more sensitive toward certain extractables, as indicated by derived EC_50_ values. The U2OS cell count is 50% compared to the control for 30 µM of *N*-Lauryldiethanolamine after 24 h incubation. In contrast, the EC_50_ of CHO cells is more than one log below that concentration (2.1 µM) after 72 h incubation. The other three compounds with an observed biological activity in the CPA (Table 1) did not impact cell growth of U2OS cells at concentrations ≤ 50 µM. When tested with different industrial cell lines, bD*t*BPP showed EC_50_ values below 1 µM, which is in agreement with the observed EC_50_ value for our CHO and other cell lines^18,58,63^. The observed differences in CPA versus CHO test results can be regarded as influenced by both cell-specific and test-specific differences, as described elsewhere^64^. The lower sensitivity of the U2OS cell line and higher test concentration needed to reduce the cell count to 50% (30 µM) in the CPA compared to the ASTM test is most likely attributed to the presence of serum in the assay^65^.

**Table 2:** EC_50_ data for CHO DG44 cells spiked with extractables.

Compound	EC50 [µM] with CHO cells
*N*-Lauryldiethanolamine	2.1
bD*t*BPP	0.60
4-*n*-Nonylphenol	No inhibition at concentrations up to 4.5
TPO	4.4

Three of the four compounds active in the CPA also negatively impact cell growth on CHO cells, showing that the ASTM test is highly sensitive for cell growth (one endpoint/feature) using host cell lines. However, the CPA provides the best conditions to detect biological effects (multiple features) in human cells﻿.

### Comparison of CPA results with toxicological screening

All results of the QSAR screening are presented in detail in SI-Table 3. Note that in vitro genotoxicity data was not checked for in vivo relevance by a weight of evidence approach, which would require the inclusion of further study data. Only 12 of 45 extractables were without any QSAR alert, which indicates from the author’s point of view that the QSAR models are very conservative. Therefore, compounds with less than two QSAR alerts were not reported in Table 3. Compounds with positive in vitro data or multiple QSAR alerts were highlighted as “substances of concern” (SI-Table 3). The results of QSAR analysis and in vitro genotoxicity studies were compared with the results of the CPA (Table 3). As outcome, compounds with toxicological alerts are not necessarily active in the CPA and vice versa. The CPA provides new data which are neither covered by QSAR analysis nor by already established test systems, such as USP 〈87〉 and ISO 10993-5 studies or in vitro mutagenicity studies. The biological results were not predictable with the toxicological assessments obtained by the applied QSAR analysis tools. Two of the four compounds with biological effects in the CPA and only one of the three compounds active in the cell growth assay showed an alert in the in silico toxicological assessment. The compound bD*t*BPP even has a low toxic hazard classification with Cramer class I. The newly identified *N*-Lauryldiethanolamine has a high toxic hazard classification with Cramer class III. It is used as additive for polymers and its presence should be excluded in processes utilizing cells.

**Table 3:** In silico toxicological assessment of compounds with at least two alerts.

Compound	Cramer class ^a^	Vega consensus model on mutagenicity (consensus score)	From QSAR-toolbox	
			Risk prediction QSAR models	Risk based on in vitro genotoxicity data from database
*N*-Lauryldiethanolamine^b^	III	Non-mutagenic (0.75)	No alert	No study data available
bD*t*BPP^b^	I	Non-mutagenic (0.675)	No alert	No data
4-n-Nonylphenol^b^	II	Non-mutagenic (1)	Alert for Michael addition	No study data available
TPO^b^	III	Mutagenic (0.1)	Alert, Schiff base formation	No alert
Caprolactam^c^	III	Non-mutagenic (1)	Alert for aclyation	Positive in vitro data
1-Acetylpyrrolidin-2-one	III	Non-mutagenic (0.5)	Multiple alerts	No data
2,4-Di-*tert*-butylphenol	I	Non-mutagenic (0.9)	No alerts	Positive in vitro data
2,6-di-*tert*-butyl-1,4-benzoquinone	III	Non-mutagenic (0.3)	Multiple alerts, high risk profile	No data
3,3'-Dinitrobisphenol A	III	Mutagenic (0.2)	Multiple alerts, high risk profile	No data
4-Hydroxy-1-(2-hydroxyethyl)-2,2,6,6-tetramethylpiperidine	III	Mutagenic (0.2)	Multiple alerts	No data
7,9-Di-*tert*-butyl-1-oxaspiro(4,5)deca-6,9-diene-2,8-dione	III	Non-mutagenic (0.35)	Multiple alerts	No data
Acetophenone	I	Non-mutagenic (1)	No alerts	Positive in vitro data
Aniline	III	Non-mutagenic (1)	Alert, nucleophilic substitution	Positive in vitro data
Benzylalcohol	I	Non-mutagenic (1)	Multiple alerts	Positive in vitro data
Bisphenol A	III	Non-mutagenic (1)	Alert, Michael addition	Positive in vitro data
Butylated hydroxytoluene	II	Non-mutagenic (1)	Alert for Michael addition	Positive in vitro data
1,3:2,4-Bis(3,4-dimethylobenzyl(ideno) sorbitol	III	Non-Mutagenic (0.2)	Multiple alerts	No data
2-Mercaptobenzothiazole	III	Non-mutagenic (0.25)	No alerts	Positive in vitro data
Phenol	III	Non-mutagenic (1)	No alerts	Positive in vitro data

^a^Toxic hazard classification by Cramer (extended): Class I = low, Class II = medium, Class III = high; ^b^Active in the CPA .

## Summary and conclusion

This feasibility study highlights the importance of comprehensive biocompatibility testing for SUS in biopharmaceutical and ATMP manufacturing. The CPA and cytotoxicity screening conducted on the human U2OS, and CHO cell lines provided valuable insights into the biological effects of plastic-related chemicals, particularly extractables that could potentially leach into pharmaceutical products.

The results showed that most of the 45 tested extractables did not significantly affect the morphological features of the cells at concentrations relevant to SUS. However, a subset of compounds, including *N*-Lauryldiethanolamine, bis(2,4-di-*tert*-butylphenyl)phosphate (bDtBPP), 4-n-nonylphenol, and diphenyl(2,4,6-trimethylbenzoyl)phosphine oxide (TPO), demonstrated biological activity in the CPA. These findings were further supported by cytotoxicity testing with CHO cells, which confirmed the potential risks of three of these compounds. QSAR tools were used to screen for toxicological alerts, such as mutagenicity, revealing that results from QSAR analysis do not always correlate with the observed biological activity. This highlights the need for a combination of in vitro and in silico methods for a comprehensive assessment. Consequently, compounds identified to show cytotoxic effects in in vitro test assays should be avoided or, at least, their quantity should be reduced and controlled in plastics used for SU equipment or ancillary materials in ATMP manufacturing^62^.

In conclusion, the integration of CPA with existing tools, such as cytotoxicity testing and QSAR analysis, offers a promising approach to support the evaluation of the biocompatibility of materials used in SUS. The findings emphasize the necessity for careful selection and qualification of single-use devices to ensure patient safety and product quality in biopharmaceutical and ATMP processes. The tested compounds should be extended for extractables or extracts of other contact materials which were not part of this feasibility study, for example polyvinyl chloride (PVC) used as material for tubing in ATMP processes.

The study also shows the need for the continued development and refinement of testing methods to better predict and mitigate the risks associated with process equipment-related leachables. This includes creating realistic exposure scenarios in ATMP processes for both cells and patients^13^. To establish a toxicity screening test based on the CPA, a set of tests comprising various differentiated cell lines is required^18^. These test scenarios should cover not only common cell features but also specific cell features that are relevant for differentiated cells in ATMP. However, this approach goes beyond the scope of this feasibility study.

### Supplementary Information

Supplementary Information 1.

Supplementary Information 2.

## Data Availability

The dataset used and/or analyzed during the current study are available from the corresponding author on reasonable request.

## References

[1] Shukla AA, Gottschalk U (2013). Single-use disposable technologies for biopharmaceutical manufacturing. Trends Biotechnol..

[2] Lopes AG (2015). Single-use in the biopharmaceutical industry: A review of current technology impact, challenges, and limitations. Food Bioprod. Process..

[3] FDA. U.S. Food and Drug Administration, 21CFR221.65 - Equipment Construction. (2017).

[4] United State Pharmacopoeia 〈665〉 Plastic Components and Systems Used in the Manufacturing of Pharmaceutical and Biopharmaceutical Drug Products (2022).

[5] United State Pharmacopoeia 〈1663〉 Assessment of Extractables Associated with Pharmaceutical Packaging/Delivery Systems. **41**, 7910–7924 (2018).

[6] Li K (2015). Creating a holistic extractables and leachables (E&L) program for biotechnology products. PDA J. Pharm. Sci. Technol..

[7] Aysola, M. *et al.* BPSA - Extractables/Leachables Considerations for Cell & Gene Therapy Drug Product Development. *Bio-Process Syst. Alliance* **17** (2020).

[8] United States Pharmacopeia 〈1043〉 - Ancillary Materials for Cell, Gene, and Tissue-Engineered Products. 10.31003/USPNF_M620_02_01 (2022)

[9] Atouf F, Provost NM, Rosenthal FM (2013). Standards for ancillary materials used in cell- and tissue-based therapies. Bioprocess Int..

[10] Arroyo A (2024). Cell and gene therapies: Challenges in designing extractables and leachables studies and conducting safety assessments. J. Pharm. Sci..

[11] Pahl I (2018). Development of a standardized extractables approach for single-use components - general considerations and practical aspects. Bioprocess Int..

[12] Scott B (2020). BioPhorum best practice guide for: Extractables testing of polymeric single-use components used in biopharmaceutical manufacturing. BioPhorum.

[13] Bossong M (2023). Biosorption of process-equipment-related leachables (PERLs) in biomanufacturing: A quantitative approach to study partitioning of PERLs in a cell culture system. Int. J. Pharm..

[14] Paudel K, Hauk A, Maier T, Menzel R (2020). Quantitative characterization of leachables sinks in biopharmaceutical downstream processing. Eur. J. Pharm. Sci..

[15] Jenke D (2022). Extractables and Leachables: Characterization of Drug Products, Packaging, Manufacturing and Delivery Systems, and Medical Devices.

[16] Hauk A, Jurkiewicz E, Pahl I, Loewe T, Menzel R (2018). Filtration membranes - scavengers for leachables?. Eur. J. Pharm. Sci..

[17] Magarian N, Lee K, Nagpal K, Skidmore K, Mahajan E (2016). Clearance of extractables and leachables from single-use technologies via ultrafiltration/diafiltration operations. Biotechnol. Prog..

[18] Budde D, Jurkiewicz E (2021). Risk analysis of leachables in cell and gene therapy using a CAR-T model process. Int. J. Pharm..

[19] Bailey MA (2012). Balancing tissue and tumor formation in regenerative medicine. Sci. Transl. Med..

[20] United State Pharmacopoeia 〈87〉 Biological Reactivity Tests, In Vitro. **43**, (2020).

[21] ISO 10993–5:2009 - Biological Evaluation of Medical Devices - Part 5: Tests for In Vitro Cytotoxicity.

[22] Eibl R (2014). Recommendations for Leachables Studies: Standardized Cell Culture Test for Early Identification of Critical Films.

[23] ASTM E3231–19: Standard Guide for Cell Culture Growth Assessment of Single-Use Material. ASTM International https://www.astm.org. 10.1520/E3231-19 (2019).

[24] Combes R (1999). Cell transformation assays as predictors of human carcinogenicity. Altern. Lab. Anim..

[25] Knight A, Bailey J, Balcombe J (2006). Animal carcinogenicity studies: 3. Alternatives to the bioassay. Altern. Lab. Anim..

[26] United State Pharmacopoeia 〈88〉 Biological Reactivity Tests, In Vivo. **43**, (2020).

[27] Directive 2010/63/EU of the European Parliament and of the Council of 22 September 2010 on the Protection of Animals Used for Scientific Purposes. *Off. J. Eur. Union* (2010).

[28] Guideline on the Principles of Regulatory Acceptance of 3Rs (Replacement, Reduction, Refinement) Testing Approaches. (2014).

[29] Broschard TH (2016). Assessing safety of extractables from materials and leachables in pharmaceuticals and biologics – current challenges and approaches. Regul. Toxicol. Pharmacol..

[30] European Medicines Agency. European Medicines Agency (EMA) - ICH Guideline M7(R1) on Assessment and Control of DNA Reactive (Mutagenic) Impurities in Pharmaceuticals to Limit Potential Carcinogenic Risk (Step 5). **44** (2018).

[31] International Council for Harmonization (ICH): Q3C (R6) - Guideline for Residual Solvents. (2019).

[32] International Conference on Harmonisation (ICH): Q3D (R2) Elemental Impurities Guidance for Industry. (2022).

[33] International Council for Harmonization (ICH): Q3C (R6) on Impurities - Support Document 2: Toxicological Data for Class 2 Solvents. *EMA* (2018).

[34] European Medicines Agency - Guideline on Setting Health-Based Exposure Limits for Use in Risk Identification in the Manufacture of Different Medicinal Products in Shared Facilities. **44** (2014).

[35] ISO 10993-17:2009 - Biological Evaluation of Medical Devices - Part 17: Establishment of Allowable Limits for Leachable Substances. 10.12455/j.issn.1671-7104.240016 39993989

[36] Kamuf J (2018). Oleic acid-injection in pigs as a model for acute respiratory distress syndrome. JoVE.

[37] Oleic acid [MAK Value Documentation, 2002]. in *The MAK‐Collection for Occupational Health and Safety*. 10.1002/3527600418.mb11280kske0017 (2012).

[38] Tobiassen, L. S., Nielsen, E., Nørhede, P. & Ladefoged, O. *Report on the Health Effects of Selected Pesticide Coformulants*. *Pesticides Research no. 80* (2003).

[39] ISO 10993-2:2022 - Biological Evaluation of Medical Devices - Part 2: Animal Welfare Requirements.

[40] Test No. 442E: In Vitro Skin Sensitisation. (OECD, 2022). 10.1787/9789264264359-en.

[41] Severin Y (2024). Multiplexed high-throughput immune cell imaging reveals molecular health-associated phenotypes. Sci. Adv..

[42] Schneidewind T (2020). Morphological profiling identifies a common mode of action for small molecules with different targets. ChemBioChem.

[43] Bray MA (2016). Cell painting, a high-content image-based assay for morphological profiling using multiplexed fluorescent dyes. Nat. Protoc..

[44] Pahl A (2023). Morphological subprofile analysis for bioactivity annotation of small molecules. Cell Chem. Biol..

[45] Ljosa V (2013). Comparison of methods for image-based profiling of cellular morphological responses to small-molecule treatment. J. Biomol. Screen..

[46] Schneidewind T (2021). Combined morphological and proteome profiling reveals target-independent impairment of cholesterol homeostasis. Cell Chem. Biol..

[47] Reisen F (2015). Linking phenotypes and modes of action through high-content screen fingerprints. Assay Drug Dev. Technol..

[48] Seal S (2023). Merging bioactivity predictions from cell morphology and chemical fingerprint models by leveraging similarity to training data. J. Cheminform..

[49] Berg EL (2019). Human cell-based in vitro phenotypic profiling for drug safety-related attrition. Front. Big Data.

[50] Rohban MH (2017). Systematic morphological profiling of human gene and allele function via cell painting. Elife.

[51] De Castelbajac T (2023). Innovative tools and methods for toxicity testing within PARC work package 5 on hazard assessment. Front. Toxicol..

[52] Nyffeler J (2020). Bioactivity screening of environmental chemicals using imaging-based high-throughput phenotypic profiling. Toxicol. Appl. Pharmacol..

[53] Alijagic A (2023). A novel nanosafety approach using cell painting, metabolomics, and lipidomics captures the cellular and molecular phenotypes induced by the unintentionally formed metal-based (nano)particles. Cells.

[54] Woehrmann MH (2013). Large-scale cytological profiling for functional analysis of bioactive compounds. Mol. Biosyst..

[55] Christoforow A (2019). Design, synthesis, and phenotypic profiling of pyrano-furo-pyridone pseudo natural products. Angew. Chem. Int. Ed..

[56] Grigalunas M (2021). Natural product fragment combination to performance-diverse pseudo-natural products. Nat. Commun..

[57] Kötzner L (2016). The organocatalytic approach to enantiopure 2H- and 3H-pyrroles: Inhibitors of the hedgehog signaling pathway. Angew. Chem. Int. Ed..

[58] Budde D (2020). Identification and evaluation of cell-growth-inhibiting bDtBPP-analogue degradation products from phosphite antioxidants used in polyolefin bioprocessing materials. Anal. Bioanal. Chem..

[59] Lapenna S, Worth A (2011). Analysis of the Cramer classification scheme for oral systemic toxicity - implications for its implementation in Toxtree (EUR 24898 EN). JRC Sci. Tech. Rep. EUR.

[60] Villamil Giraldo AM, Appelqvist H, Ederth T, Öllinger K (2014). Lysosomotropic agents: Impact on lysosomal membrane permeabilization and cell death. Biochem. Soc. Trans..

[61] Nadanaciva S (2011). A high content screening assay for identifying lysosomotropic compounds. Toxicol. Vitr..

[62] Jurkiewicz E, Husemann U, Greller G, Barbaroux M, Fenge C (2014). Verification of a new biocompatible single-use film formulation with optimized additive content for multiple bioprocess applications. Biotechnol. Prog..

[63] Jachuck JR, Krishnathu SM, Landau JE, Ko HF, Bhatia R (2020). Sensitivity of a PER.C6® cell line to bis(2,4-di-tert-butylphenyl)phosphate and evaluation of a new biocompatible single-use film. Biotechnol. Prog..

[64] Rietdijk J (2022). Morphological profiling of environmental chemicals enables efficient and untargeted exploration of combination effects. Sci. Total Environ..

[65] Jurkiewicz, E. & Tappe, A. Assessing Cell Lines for Cell Growth Assays as an Alternative to Existing Cytotoxicity Assays. in *Single-Use Technologies II: Bridging Polymer Science to Biotechnology Applications* (eds. Mahajan, E. & Lye, G.) (ECI Symposium Series, 2015).

