# Supplementary material for: Assessing biologic/toxicologic effects of extractables from plastic contact materials for advanced therapy manufacturing using cell painting assay and cytotoxicity screening
Source: Sci Rep. 2024 Mar 11;14:5933. doi: 10.1038/s41598-024-55952-3 (PMC10928227; doi:10.1038/s41598-024-55952-3)
Supplement: Supplementary file 1 — Supplementary Information 1. [file 41598_2024_55952_MOESM1_ESM.docx]

Supporting Information Assessing Biologic/Toxicologic Effects of Extractables from Plastic Contact Materials for Advanced Therapy Manufacturing Using Cell Painting Assay and Cytotoxicity Screening

**SI-Table 1: Selection of Tests for the Qualification of SU contact materials for Cell and Gene Therapy (no claim to comprehensiveness).**

| Category | Test | Test Principle | Output | Validity regarding Patients´ Safety |
| --- | --- | --- | --- | --- |
| **Experimental Methods** | | | | |
| Elastomeric plastics and other polymeric materials with patient contact | USP 〈87〉 | *In vitro* | Biological reactivity on cells | Results are not directly transferable, different sensitivity of cells are to be taken into account |
|  | USP 〈88〉 | *In vivo* | Biological response of animals | Only indicative |
| Single-Use material in biopharma applications | Standard Guide for Cell Culture Growth Assessment of Single-Use Material | *In vitro* | Compatibility of material extracts in biopharmaceutical manufacturing processes with host cells | ASTM E3231 – 19; relevant for process performance |
|  | Recommendation for Leachables Studies Standardized Cell Culture Test for the Early Identification of Critical Films | *In vitro* | Biocompatibility of film materials with host cells (only CHO) | DECHEMA recommendation relevant for process performance; only for CHO; similar to ASTM E3231 – 19 |
| Material for medical devices | ISO 10993-3 | *In vivo* | Genotoxicity, carcinogenicity, and reproductive toxicity | Specifc design of the tests is required in order to the get an adequate exposure of target cells/tissue |
|  | ISO 10993-4 | *In vivo; in vitro* | Interactions with blood | Relevant for effects on the blood system |
|  | ISO 10993-5 | *In vitro* | Cytotoxicity | Results are not directly transferable, different sensitivity of cells are to be taken into account |
|  | ISO 10993-6 | *In vivo* | Local effects after implantation | Results are not directly transferable, different sensitivity of tissues and cells are to be taken into account |
|  | ISO 10993-11 | *In vivo* | Systemic toxicity | Specifc design of the tests is required in order to the get an adequate exposure of target cells/tissue |
|  | Bacterial Reverse Mutation Test (“Ames Test”) | *In vitro, w/wo activation* | Mutagenicity: point mutations by base substitutions or frameshifts | OECD 471; usefuld for the endpoint “mutagenicity” in an integrated testing strategy for a product or a substance |
|  | *In vitro* Mammalian Chromosomal Aberration Test | *In vitro* | Mutagenicity: structural chromosome aberrations in cultured mammalian somatic cells | OECD 473; maybe useful for the endpoint “mutagenicity” in an integrated testing strategy for a product or a substance |
|  | Mammalian Bone Marrow Chromosomal Aberration Test | *In vivo* | Structural chromosome aberrations induced by test compounds in bone marrow cells of rodents | OECD 475; maybe useful for the endpoint “mutagenicity” in an integrated testing strategy for a product or a substance |
|  | *In vitro* Mammalian Cell Micronucleus Test | *In vitro* | Genotoxicity test: detection of micronuclei in the cytoplasm of interphase cells | OECD 487; maybe useful for the endpoint “mutagenicity” in an integrated testing strategy for a product or a substance |
|  | *In vitro* Mammalian Cell Gene Mutation Test | *In vitro* | Gene mutation: detection of gene mutations | OECD 476; maybe useful for the endpoint “mutagenicity” in an integrated testing strategy for a product or a substance |
|  | Mammalian Erythrocyte Micronucleus Test | *In vivo* | Damage to the chromosomes or the mitotic apparatus of erythroblasts in rodents | OECD 474; maybe useful for the endpoint “mutagenicity” in an integrated testing strategy for a product or a substance |
|  | Transgenic Rodent Somatic and Germ Cell Gene Mutation Assays | *In vivo* | Gene mutations in somatic and germ cells in transgenic mouse | OECD 488; maybe useful for the endpoint “mutagenicity” in an integrated testing strategy for a product or a substance |
|  | Repeated Dose 28-Day Oral Toxicity Study in Rodents | *In vivo* | For hazard identification and risk assessment | OECD 407; results are not directly transferable, route-to-route application may be disputable |
|  | Repeated dose 90-day oral toxicity study in rodents | *In vivo* | Estimate of a no-observed-adverse-effect level | OECD 408; results are not directly transferable, route-to-route application may be disputable |
|  | Carcinogenicity Studies | *In vivo* | Carcinogenicity in rodents | OECD 451; results are not directly transferable, route-to-route application may be disputable |
| **In silico QSAR tools for toxicity assessment** | | | | |
| Compounds | Toxtree | *In silico* | Toxicological parameters: e.g. (non) genotoxicity, mutagenicity | <http://toxtree.sourceforge.net>; substance specific; rule-based structure analysis; non-commercial |
|  | Vega | *In silico* | Toxicological parameters: e.g.,carcinogenicity; mutagenicity | <https://www.vegahub.eu/>; substance specific; property prediction using different QSAR models, non-commercial |
|  | QSAR Toolbox 4.5 | *In silico* | Toxicological parameters: e.g.,carcinogenicity; mutagenicity, genotoxicity | <https://qsartoolbox.org>; substance specific; property prediction using different QSAR models and read across, non-commercial |

SI-Table 2: Overview of tested extractable compounds and their typical polymeric sources and functionality

| Compound | CAS | Sources \| Materials | Function \| Interpretation | Ref |
| --- | --- | --- | --- | --- |
| 12-Aminododecanolactam (Laurolactam) | 947-04-6 | Polyamides | Monomer | ^1,2^ |
| 1,3:2,4-Bis(3,4-dimethylobenzyl(ideno)sorbitol | 135861-56-2 | Polyolefins | Nucleating agent | ^3,4^ |
| 1,3-Di-*tert*-butylbenzene | 1014-60-4 | Polyolefins | NIAS; Degradation product of EP plastic additive 12 | ^5,6^ |
| 1,4-Cyclohexane dimethanol | 105-08-8 | Polyester; Polyurethan | Monomer | ^7,8^ |
| 1-Acetyl-2-pyrrolidone | 932-17-2 | PES membranes | Impurity; NIAS | ^1^ |
| 1-Dodecanol | 112-53-8 | Polyolefins | NIAS; Degradation product of EP plastic additive 16 | ^1^ |
| 2 – Mercaptobenzo thiazole | 149-30-4 | Rubber | Accelerator of sulfur vulcanization of rubbers | ^9^ |
| 2 Pentanone | 107-87-9 | Polyolefins | NIAS; Degradation product of polyolefins | ^10^ |
| 2,4-Di-*tert*-butylphenol | 96-76-4 | Polyolefins | NIAS; Degradation product of EP plastic additive 12 | ^10^ |
| 2,6-di-*tert*-butyl-1,4-benzoquinone | 719-22-2 | Polyolefins | NIAS; Degradation product of EP plastic additive 9 and 11 | ^5,10^ |
| 2-Pyrrolidone | 616-45-5 | PES membrane | Solvent in membrane production | ^11^ |
| 3-(3,5-Di-*tert*-butyl-4-hydroxyphenyl) propionic acid | 20170-32-5 | Polyolefins | NIAS; Degradation product of EP plastic additive 9 and 11 | ^12^ |
| 3,3’-Dinitrobisphenol A | 5329-21-5 | Polycarbonate | NIAS; Impurity | ^13^ |
| 3,4-Dimethylbenzaldehyde | 5973-71-7 | Polyolefins | Degradant of clarifying agent | ^14^ |
| 3,5-Di-*tert*-butyl-4-hydroxybenzaldehyde | 1620-98-0 | Polyolefins | NIAS; Degradation product of EP plastic additive 9 and 11 | ^15^ |
| 4-Hydroxy-1-(2-hydroxyethyl)-2,2,6,6-tetramethylpiperidine | 52722-86-8 | Polyolefins | NIAS; Degradation of HALS; EP plastic additive 22 (Tinuvin 625) | ^16^ |
| 4-Methyl benzaldehyde | 104-87-0 | Polyolefins | NIAS; Degradant of nucleating agent | ^17^ |
| 4-n-Nonylphenol | 104-40-5 | Polyolefins | EP plastic additve 18; Plastizicer | ^18,19^ |
| 7,9-Di-*tert*-butyl-1-oxaspiro(4,5)deca-6,9-diene-2,8-dione | 82304-66-3 | Polyolefins | NIAS; Degradation product of EP plastic additive 9 and 11 | ^11^ |
| Acetophenone | 98-86-2 | Elastomers; ABS | NIAS; Degradation product | ^6^ |
| Aniline | 62-53-3 | Starting material for modification of membrane surfaces | Solvent | ^20^ |
| Benzyl alcohol | 100-51-6 | PVC, Elastomers | Solvent | ^6^ |
| Bis(2-hydroxyethyl)terephthalate | 959-26-2 | PET | NIAS; Degradant of PET in Ethanol extracts | ^21^ |
| Bis(2,4-di-t*ert*-butylphenyl)phosphate (bD*t*BPP) | 69284-93-1 | Polyolefins | NIAS; Degradation product of EP plastic additive 12 | ^22^ |
| Bis-(4-chlorphenyl)-sulfon | 80-07-9 | PES membranes | Monomer | ^23^ |
| Bisphenol A (BPA) | 80-05-7 | Polycarbonate | Monomer | ^24,25^ |
| Butylhydroxytoluene (BHT) | 128-37-0 | Polyolefins | Antioxidant: EP plastic additive 7 | ^3^ |
| Caprolactam | 105-60-2 | PES; Polyamide | Solvent; Monomer | ^1,2,26^ |
| Dibutylphthalate | 84-74-2 | PVC, Polyolefins | Plasticizer | ^27^ |
| Diphenyl carbonate | 102-09-0 | Polycarbonate | Monomer | ^28^ |
| Diphenyl(2,4,6-trimethylbenzoyl)phosphine oxide (TPO) | 75980-60-8 | Polyesters; Polyacrylic esters | Photo initiator | ^29^ |
| Ethyl 4-ethoxybenzoate | 23676-09-7 | Polyethylenes | Donor in catalyst for polymerization of polyethylene | ^23^ |
| Glycerol | 56-81-5 | Cellulose Acetate and Derivates | Degradant plastic additives glycerol esters; Hydrophilization agent | ^23^ |
| Hexanal | 66-25-1 | Polypropylene | NIAS; Degradant after irradiation | ^30^ |
| Methyl ethyl ketone | 78-93-3 | Polyolefins | Solvent; Degradant after irradiation | ^31^ |
| *N*-Butylbenzenesulfonamide | 3622-84-2 | Polyurthane, polyamide | Plasticizer | ^32^ |
| *N*-Lauryldiethanolamine | 1541-67-9 | Polyolefins | Antistatic agent | ^3^ |
| *N*-Methyl-2-pyrrolidone | 872-50-4 | PES membranes | Solvent | ^23^ |
| Octamethyl cyclotetrasiloxane (D4) | 556-67-2 | Silicones | Monomer and\|or reaction by-product | ^33,34^ |
| Octanoic acid | 124-07-2 | Polyolefins | Starting agent; emulsifier | ^6^ |
| Phenol | 108-95-2 | PVC | Polymerization aid | ^6^ |
| p-Toluenesulfonamide | 70-55-3 | PVC | Plasticizer | ^32^ |
| PVP-Copolymer | 25086-89-9 | PES membranes | Hydrophilization agent | ^1,35^ |
| PVP after gamma irradiation | 25086-89-9_g | PES membranes | Hydrophilization agent | ^35^ |
| *tert*-Butanol | 75-65-0 | Polyolefins | NIAS; Degradation product of polyethylene | ^10^ |
| Ethanol extract of PES filter cartridge, such as PVP-coplymer, PES oligomers, Irgafos^®^ 168, Irganox^®^ PS 800 and its degradants | n.a. | Poleolefins, PES, PET | Ethanol extract from 0.2 µm-rated PES membrane filter cartridge: 24 h at 40°C with effective filtration area to volume ratio 1 cm^2^/mL. | ^17,36^ |
| 50% Ethanol extract of silicone tubings, such as siloxane oligomers | n.a. | Silicone | 50% Ethanol/water extract (v:v) from silicone tube: 21 days at 40°C with surface area to volume ratio 6 cm^2^/mL. | ^37^ |

n.a. not applicable; NIAS: Non-intentionally added substance; ABS: Acrylonitrile butadiene styrene; PES: polyethersulfone; PET: polyethylene terephthalate; PVC: Polyvinyl chloride; PVP: polyvinylpyrrolidone.

**Rational Compound Selection**

The selected molar concentration range for the CPA is 10 to 50 µM, which corresponds to extractables mass concentrations between 0.7 µg/mL and 23.7 µg/mL. This range exceeds the commonly observed mass concentrations of extractables obtained under worst-case extraction conditions (between 0.1 and 10 µg/mL).^1,17,38^ They include compounds that can be released by plastics used in SUT or laboratory equipment, such as well plates. These consist of a) intentionally added plastic additives relevant to the functionality and performance of the plastic, e.g., antioxidant butylhydroxytoluene (BHT, CAS 128-37-0) and clarifying agent dimethyldibenzylidene sorbitol (CAS 135861-56-2), b) polymer monomers, e.g., bisphenol A (BPA) for polycarbonate and laurolactam (CAS 947-04-6) for polyamide PA12, and c) degradants of the additives or the polymer, such as dodecanol (CAS 112-53-89, 7,9-di-tert-butyl-1-oxaspiro(4,5)deca-6,9-diene-2,8-dione (CAS 82304-66-3), or bis(2-hydroxyethyl)terephthalate (CAS 959-26-2). Degradants are typical non-intentionally added substances (NIAS). They can be formed after oxidation or breakdown due to sterilization of the devices (irradiation). It is worth mentioning that only very few plastic-related compounds have been reported to show a detrimental effect on cells, such as bis(2,4-di-tert-butylphenyl)phosphate (bDtBPP) and 3,3’-dinitrobisphenol A,^13,39,40^ or are a risk to the patient, e.g., due to sensitizing effects (2-Mercaptobenzothiazol). These compounds are not expected to be released at relevant levels from elastomers and plastics used today but have been included in the selected test compounds. Finally, the CPA was used to test extracts from an extraction study of two SU components – a sterilizing-grade filter and silicone tubing. Details on extractions and extractables are given in SI-Table 2.

Almost all compounds tested have a log P ≤ 5. Such compounds could be present in the aqueous AT process media containing buffer salts, proteins, surfactants, or other excipients. The compound bDtBPP has a calculated log P value of 8.4 and is known detrimental effect on cell growth (EC50 value for CHO cells is < 1 µM).^22^ It is also slightly soluble in water due to the deprotonation of the phosphate. The compound bDtBPP is considered as relevant to challenge the capabilities of the CPA in order to detect compounds which are known to show a detrimental effects on cell growth.

Results of the QSAR screening are presented in a separate Excel file in SI Table 3

**References**

1. Menzel, R., Pahl, I., Loewe, T. & Hauk, A. Comparative Extractables Study of Autoclavable Polyethersulfone Filter Cartridges for Sterile Filtration. *PDA J. Pharm. Sci. Technol.* **72**, 298–316 (2018).

2. 21CFR177.1500 - Nylon resins.

3. Zweifel, H., Maier, R. D. & Schiller, M. *Plastics Additives Handbook*. (Carl Hanser Verlag GmbH & Co. KG, 2009).

4. 21CFR178.3295 - Clarifying agents for polymers.

5. Makhzoumi, Z. El. Effect of irradiation of polymeric packaging material on the formation of volatile compounds. in *Food Packaging and Preservation* 88–99 (Springer US, 1994). doi:10.1007/978-1-4615-2173-0_5.

6. Jenke, D. *Compatibility of Pharmaceutical Products and Contact Materials - Appendix: Materials Used in Pharmaceutical Constructs and their Associated Extractables*. (John Wiley & Sons, Inc., 2009).

7. 21CFR177.1240 - 1,4-Cyclohexylene dimethylene terephthalate and 1,4-cyclohexylene dimethylene isophthalate copolymer.

8. 21CFR177.1315 - Ethylene-1, 4-cyclohexylene dimethylene terephthalate copolymers.

9. Umweltbundesamtes, B. des. Stoffmonographie für 2-Mercaptobenzothiazol (2-MBT) und HBM-Werte für 2-MBT im Urin von Erwachsenen und Kindern. *Bundesgesundheitsblatt - Gesundheitsforsch. - Gesundheitsschutz* **58**, 1027–1040 (2015).

10. Kawamura, Y. Effects of Gamma Irradiation on Polyethylene, Polypropylene, and Polystyrene. in *Irradiation of Food and Packaging* 262–276 (American Chemical Society, 2004). doi:10.1021/bk-2004-0875.ch016.

11. Menzel, R., Pahl, I., Loewe, T. & Hauk, A. Comparative Extractables Study of Autoclavable Polyethersulfone Filter Cartridges for Sterile Filtration. *PDA J. Pharm. Sci. Technol.* **72**, 298–316 (2018).

12. Buchalla, R., Schuettler, C. & Boegl, K. W. Effects of Ionizing Radiation on Plastic Food Packaging Materials: A Review - Part 2. *J. Food Prot.* **56**, 998–1005 (1993).

13. Peng, J. *et al.* Chemical Identity and Mechanism of Action and Formation of a Cell Growth Inhibitory Compound from Polycarbonate Flasks. *Anal. Chem.* **90**, 4603–4610 (2018).

14. McDonald, J. G., Cummins, C. L., Barkley, R. M., Thompson, B. M. & Lincoln, H. A. Identification and Quantitation of Sorbitol-Based Nuclear Clarifying Agents Extracted from Common Laboratory and Consumer Plasticware Made of Polypropylene. *Anal. Chem.* **80**, 5532–5541 (2008).

15. Nieva-Echevarría, B., Manzanos, M. J., Goicoechea, E. & Guillén, M. D. 2,6-Di-Tert-Butyl-Hydroxytoluene and Its Metabolites in Foods. *Compr. Rev. Food Sci. Food Saf.* **14**, 67–80 (2014).

16. Reisinger, M., Beißmann, S. & Buchberger, W. Quantitation of hindered amine light stabilizers in plastic materials by high performance liquid chromatography and mass spectrometric detection using electrospray ionization and atmospheric pressure photoionization. *Anal. Chim. Acta* **803**, 181–187 (2013).

17. Pahl, I., Menzel, R., Hauk, A. & Loewe, T. Using Extractables Data of Sterile Filter Components for Scaling Calculations. *PDA J. Pharm. Sci. Technol.* **73**, 523–537 (2019).

18. Celiz, M. D., Morehouse, K. M., DeJager, L. S. & Begley, T. H. Concentration changes of polymer additives and radiolysis products in polyethylene resins irradiated at doses applicable to fresh produce. *Radiat. Phys. Chem.* **166**, 108520 (2020).

19. Jenke, D. *et al.* Simulated Leaching (Migration) Study for a Model Container-Closure System Applicable to Parenteral and Ophthalmic Drug Products. *PDA J. Pharm. Sci. Technol.* **71**, 68–87 (2017).

20. European Medicines Agency. European Medicines Agency (EMA) - ICH Guideline M7(R1) on Assessment and Control of DNA Reactive (Mutagenic) Impurities in Pharmaceuticals to Limit Potential Carcinogenic Risk (Step 5). **44**, (2018).

21. Besnoin, J.-M. & Choi, K. Y. Identification and characterization of reaction byproducts in the polymerization of polyethylene terephthalate. *J. Macromol. Sci. Part C* **29**, 55–81 (1989).

22. Hammond, M. *et al.* Identification of a leachable compound detrimental to cell growth in single-use bioprocess containers. *PDA J. Pharm. Sci. Technol.* **67**, 123–34 (2013).

23. Commission Regulation (EU) No 10/2011 on plastic materials and articles intended to come into contact with food. (2011).

24. Biedermann-Brem, S., Grob, K. & Fjeldal, P. Release of bisphenol A from polycarbonate baby bottles: mechanisms of formation and investigation of worst case scenarios. *Eur. Food Res. Technol.* **227**, 1053–1060 (2008).

25. Sajiki, J. & Yonekubo, J. Leaching of bisphenol A (BPA) from polycarbonate plastic to water containing amino acids and its degradation by radical oxygen species. *Chemosphere* **55**, 861–867 (2004).

26. Bonifaci, L., Frezzotti, D., Cavalca, G., Malaguti, E. & Ravanetti, G. P. Analysis of ε-caprolactam and its cyclic oligomers by high-performance liquid chromatography. *J. Chromatogr. A* **585**, 333–336 (1991).

27. ECHA - Support Document to the Opinion of the Member State Committee for Identification of Dibutyl Phthalate (DBP). at (2014).

28. Brydson, J. A. Polycarbonates. in *Plastics Materials* (ed. Brydson, J. A. B. T.-P. M. (Seventh E.) 556–583 (Butterworth-Heinemann, 1999). doi:https://doi.org/10.1016/B978-075064132-6/50061-9.

29. *Scientific Committee on Consumer Safety SCCS (SCCS/1528/14) - Trimethylbenzoyl diphenylphosphine oxide (TPO)*. (2014). doi:10.2772/45370.

30. Makhzoumi, Z. El. Effect of irradiation of polymeric packaging material on the formation of volatile compounds. in *Food Packaging and Preservation* 88–99 (Springer US, 1994). doi:10.1007/978-1-4615-2173-0_5.

31. Azuma, K., Hirata, T., Tsunoda, H., Ishitani, T. & Tanaka, Y. Identification of the volatiles from low density polyethylene film irradiated with an electron beam. *Agric. Biol. Chem.* **47**, 855–860 (1983).

32. Wypych, G. *Handbook of Plasticisers*. (ChemTec Publishing, 2017).

33. ECHA. ECHA - ANNEX XV Restriction report - D4, D5 and D6. at (2019).

34. Li, K., Doland, D., Erexson, G. & Nagao, L. M. Risk Assessment of Cyclic Siloxanes (D3 to D19) as Extractables from Polymeric Components in Biopharmaceutical Manufacturing - SOT 54th Annual Meeting. at (2015).

35. Menzel, R., Pahl, I., Loewe, T., Stuetzer, A. & Hauk, A. Rinsing Recommendations for Membrane Filters and Identification of Rinsables. *Eur. J. Pharm. Sci.* **168**, 105982 (2022).

36. Menzel, R. *et al.* Structure Elucidation and Toxicological Evaluation of Cyclic Polyethersulfone Oligomers Present in Extracts of Membrane Filters. *Polym. Eng. Sci.* **62**, 2817–2825 (2022).

37. Jenke, D., Story, J. & Lalani, R. Extractables/leachables from plastic tubing used in product manufacturing. *Int. J. Pharm.* **315**, 75–92 (2006).

38. Pahl, I., Dorey, S., Barbaroux, M., Lagrange, B. & Frankl, H. Analysis and evaluation of single-use bag extractables for validation in biopharmaceutical applications. *PDA J. Pharm. Sci. Technol.* **68**, 456–71 (2014).

39. Hammond, M. *et al.* A cytotoxic leachable compound from single-use bioprocess equipment that causes poor cell growth performance. *Biotechnol. Prog.* **30**, 332–337 (2014).

40. Budde, D. *et al.* Identification and evaluation of cell- growth-inhibiting bDtBPP-analogue degradation products from phosphite antioxidants used in polyolefin bioprocessing materials. *Anal. Bioanal. Chem.* **412**, 4505–4518 (2020).
